# *M. bovis* BCG Moreau N-Terminal Loss Leads to a Less Stable Dodecin With Lower Flavin Binding Capacity

**DOI:** 10.3389/fcimb.2021.658888

**Published:** 2021-03-31

**Authors:** Marcos Gustavo Araujo Schwarz, Bianca Gallart Cinelli Luzes, Paloma Rezende Correa, Antônio José da Silva-Gonçalves, Lucas de Almeida Machado, Ana Carolina Ramos Guimarães, Leila Mendonça-Lima

**Affiliations:** ^1^ Laboratório de Genômica Funcional e Bioinformática, Instituto Oswaldo Cruz, Fiocruz, Rio de Janeiro, Brazil; ^2^ Laboratório Interdisciplinar de Pesquisas Médicas, Instituto Oswaldo Cruz, Fiocruz, Rio de Janeiro, Brazil

**Keywords:** dodecin, *Mycobacterium bovis* BCG Moreau, single nucleotide polymorphism (SNP), flavin binding protein, tuberculosis vaccine

## Abstract

Tuberculosis still remains a concerning health problem worldwide. Its etiologic agent, *Mycobacterium tuberculosis*, continues to be the focus of research to unravel new prophylactic and therapeutic strategies against this disease. The only vaccine in use against tuberculosis is based on the *in vitro* attenuated strain, *M. bovis* BCG. Dodecin is a dodecameric complex important for flavin homeostasis in Archea and Eubacteria, and the *M. tuberculosis* protein is described as thermo- and halostable. *M. bovis* BCG Moreau, the Brazilian vaccine strain, has a single nucleotide polymorphism in the dodecin start codon, leading to a predicted loss of seven amino acids at the protein N-terminal end. In this work we aimed to characterize the effect of this mutation in the BCG Moreau protein features. Our recombinant protein assays show that the predicted BCG homolog is less thermostable than *M.tb*’s but maintains its dodecamerization ability, although with a lower riboflavin-binding capacity. These data are corroborated by structural analysis after comparative modeling, showing that the predicted BCG dodecin complex has a lower interaction energy among its monomers and also a distinct electrostatic surface near the flavin binding pocket. However, western blotting assays with the native proteins were unable to detect significant differences between the BCG Moreau and *M.tb* orthologs, indicating that other factors may be modulating protein structure/function in the bacterial context.

## Introduction

Dodecin plays an important role in bacterial physiology, mainly due to its predicted function as a storage protein for cofactors needed by these organisms throughout their life cycles, such as flavins and coenzyme A (Meissner et al., 2007). In Archaea, like *Halobacterium salinarum*, this protein is suggested as a key player on flavin homeostasis, functioning as a buffer for this kind of molecule (Grininger et al., 2009), and acting as a source of active flavins (FADH_2_ and FMN) or riboflavin for production of the former when required. It may also prevent harmful effects of flavin light-induced reactions on the bacterial cytosol, similar to eukaryotic flavin-binding proteins (Kelley et al., 2017).

The Mycobacteria are known mainly for its pathogenic members, such as *M. tuberculosis* (*M.tb*) and *M. leprae*. Human tuberculosis is a worldwide spread disease, causing an estimated 1.2 million deaths among HIV-negative individuals in 2019 (World Health Organization, 2020). Dispersal of antibiotic-resistant strains and the increase in HIV coinfection cases (Lai et al., 2016) justify the urgency for new prophylactic and therapeutic approaches, warranting the need for a better understanding of mycobacterial metabolism and physiology.

The only available prophylactic method against human tuberculosis is vaccination with Bacillus Calmette-Guérin (BCG), an *in vitro* attenuated *M. bovis* strain. Factors resulting from storage and cultivation of this bacterium led to its genetic and phenotypic diversification, and today different strains are used for vaccine production worldwide (Ritz and Curtis, 2009), all part of the BCG family. Genomic studies revealed strain-specific genetic patterns, and these could be related to vaccine efficacy and other bacterial features (Brosch et al., 2007).

*M. bovis* BCG Moreau, the strain used for vaccine production in Brazil from 1925 to 2017, is closely related to other more primitive strains, such as BCG Russia and Japan, believed to be similar to the original BCG (Abdallah et al., 2015). But even with the long, continuous usage of this strain for vaccination of Brazilian children, little is known about BCG Moreau physiology, when compared to BCG Pasteur, one of the most studied BCG strains, *M. bovis* or *M.tb*. In the last decade, our group worked on its characterization, on the genomic (Gomes et al., 2011), proteomic (Berrêdo-Pinho et al., 2011; Pagani et al., 2019) and functional context (Galvão et al., 2014; Monteiro-Maia et al., 2018; Schwarz et al., 2020; Schwarz et al., 2021). Comparison of the BCG Moreau and *M.tb* genomes confirms a single nucleotide polymorphism (SNP) within the start codon of the dodecin coding gene, already annotated, but not functionally described, for BCG Pasteur (BCG_1562c), leading to a predicted N-terminal shorter homolog in BCG. Although *M.tb* dodecin is shown to be non-essential for *in vitro* growth (DeJesus et al., 2017), it may play a relevant role within the infection context, as flavins are important to the regulation and maintenance of the redox environment, making it an interesting target for drug development. Inhibitors of dodecin function could impair the normal bacterial response within the infection process, adding to the arsenal of inhibitory molecules targeting redox metabolism enzymes (Lu and Holmgren, 2014). Due to its importance in flavin homeostasis, already described for other microorganisms (Grininger et al., 2009), here we report the initial characterization of BCG Moreau dodecin, comparing it with the known *M.tb* homolog, aiming to understand the possible functional impacts of this mutation.

## Methods

### Sequence Analysis and Comparisons

*M.tb* dodecin gene sequence (GeneID: 3205040; *rv1498A*) was obtained from the public domain databank NCBI *Mycobacterium tuberculosis* H37Rv complete genome (GenBank: NC_000962.3). The *M. bovis* BCG Moreau ortholog was found by Blast analysis, identifying BCG_M1530c on the *M. bovis* BCG str. Moreau RDJ complete genome (GenBank: AM412059.2). Nucleic acid and protein sequence alignments were performed with the free online tool Clustal Omega.

### Recombinant Protein Expression, Purification and Polyclonal Sera Production

Recombinant BCG Moreau and *M.tb* dodecins (rDod), with addition of a C-terminal 6-His tag (or also without in the case of the BCG protein), were expressed in *E. coli*. *M. bovis* BCG Moreau and *M.tb* H37Rv genomic DNA were used as template to amplify the dodecin genes (BCG_M1530c and *rv1498A*, respectively) containing NcoI (5’) and XhoI (3’) restriction sites, using Platinum SuperFi DNA polymerase (Invitrogen) and the following pair of primers: AAACCATGGTGATCGAGATCGTC (forward for both BGC proteins), TGCCTCGAGGGAATCCTCCAGGCG (reverse for both BCG His-tagged and *M.tb* proteins), TTTTCTCGAGTCAGGAATCCTCCAG (reverse for BCG non-tagged protein) and AAACCATGGCGAGCAATCACACCTA (forward for *M.tb* protein).

The restriction enzyme digested and purified amplicons were cloned in pET28a(+) vector DNA digested with the same pair of enzymes. After electroporation on *E. coli* TOP10 and selection on 25 µg/mL kanamycin Luria-Bertani (LB) plates incubated at 37 °C for 16h, isolated colonies were analyzed by colony PCR and inserts were sequenced by Sanger technology using commercial pair of primers, T7 promoter and terminator.

Recombinant plasmids were transformed in *E. coli* BL21(DE3) strain and isolated colonies were cultivated on LB medium supplemented with kanamycin, at 37 °C under agitation (200 rpm). When cultures reached an optical density (O.D._600nm_) of 0.4-0.6, isopropyl-β-D-thiogalactopyranoside (IPTG) was added to 1 mM final concentration and cultures were incubated for a further 3 hours. Cells were then harvested by centrifugation, ressuspended in lysis buffer (20 mM Tris-HCl pH 7.5; 300 mM NaCl; 0.5% [v/v] Triton X-100) and disrupted by mechanical lysis on a BeadBeater equipment using glass beads and 3 one-minute cycles on ice. These induction and lysis protocols were used for all assays using rDod producing *E. coli* strains.

The resulting supernatant from the BCG His-tagged protein (rDod-BCG-6His) clone was clarified by centrifugation and 0.22 µm filtered before loading on a HisTrap IMAC HP 1mL column (GE Healthcare), charged with Ni2+. Loading buffer (buffer A) was 100 mM Tris-HCl pH 7.5, 300 mM NaCl and 5 mM imidazole, and elution was performed with elution buffer (buffer B) (same as A, but with 500 mM imidazole) by step-gradients (10, 20, 30, 40 and 100% of B, 5 column volumes [CV] each). Fractions were analyzed by 15% SDS-PAGE.

Purified fractions from rDOD-BCG-6His were pooled, dialyzed against PBS and used to immunize six BALB-C mice, with 3 x 10ug intra-peritoneal injections at 2 weeks intervals (the first two doses in Freund’s incomplete adjuvant, and the final one in sterile PBS). Whole individual mouse serum was collected and tested against purified rDOD-BCG-6His on western blotting assays to confirm antibody production. Responding sera were pooled and used as dodecin polyclonal antibody (α-dodecin). This study was approved by the Commission for the Use of Laboratory Animals from IOC, Fiocruz (License CEUA/IOC L-020/2016).

### Dodecamerization and Thermophilic In-Gel Assays

Total proteins from IPTG-induced *E. coli* cultures expressing rDods were used in these assays. Samples were resolved in 15% SDS-PAGE prior to western blotting analysis. Briefly, proteins were transferred to nitrocellulose membrane (Hybond C, GE) using a MiniTransblot equipment (BioRad) at constant 100 V for 1 hour. The quality of the protein transfer was assessed by reversible staining with Mem Code (Pierce) and the membrane blocked for 16h at 4o C in TBS-T (Tris-buffered saline supplemented with 0.05% (v/v) Tween 20) containing 10% (w/v) skim milk. After washing with TBS-T, the membrane was incubated with α-dodecin (1:100) for 2 hours, followed by incubation with HRP-conjugated goat anti-mouse IgG (1:10,000) for 1 hour. After each antibody incubation, the membrane was washed 3 times with TBS-T, followed by 3 washes with TBS. All antibody incubations were performed in TBS-T containing 5% (w/v) skim milk. Blots were developed using the SuperSignal kit (Pierce), following the manufacturer’s protocol.

For thermophilic analysis the protocol described in (Liu et al., 2011) was used, with minor modifications. Briefly, samples were incubated for 30 minutes at 25, 40 and 80°C prior to western blotting assays. After clarification by centrifugation, the same volume of supernatant was loaded on the gel and western blotting was performed to detect the presence of dodecin in solution.

### Native Dodecin In-Gel Characterization

*M.tb* H37Rv and *M. bovis* BCG Moreau cells were grown on 7H9 medium supplemented with 10% mixture of albumin, dextrose and catalase (ADC) at 37 °C under agitation (200 rpm). Cultures were started at OD_600nm_~0.1 and cells were recovered by centrifugation at days 2, 5 and 7, corresponding to lag, logarithmic and stationary phases, respectively.

Whole cell lysate was prepared by mechanical lysis on a BeadBeater equipment, using the following buffer: 50 mM HEPES/KOH pH 7.5; 10 mM MgCl2; 60 mM NH4Cl; 10% [v/v] glycerol. After clarification by centrifugation, total proteins were quantified on NanoDrop and 20 µg resolved on 15% SDS-PAGE prior to western blotting with α-dodecin, as described above.

For thermostability assays, logarithmic phase cell lysates were incubated for 30 minutes at 40, 80 or 100 °C, and the same volumes of supernatants were used as samples for western blotting.

### Riboflavin Binding Assay

Total proteins from IPTG-induced *E. coli* cultures expressing BCG or *M.tb* His-tagged rDod were used for this assay. One mL of clarified lysates, adjusted to contain equivalent protein amounts (500 ug), was loaded on 10 kDa filter units (AmiconUltra, Millipore) and the same volume of PBS was used as negative control. Samples were concentrated to around 100 µL (using the manufacture’s protocol) and washed three times with 1 mL of PBS, before addition of a saturated riboflavin solution, also in PBS. The filter units were incubated under agitation for two hours at room temperature and washed three times with PBS, in order to completely remove unbound riboflavin. Flavins have absorption peaks at 220, 265, 370 and 450 nm; to avoid protein interference, the protein-associated riboflavin remaining in the retentate was analyzed by NanoDrop at 450 nm (Liu et al., 2011) and values compared to the absorbance of the saturated riboflavin solution. Results are represented as the mean of a set of three experiments, with standard deviation, and analyzed using a non-paired t-test on GraphPad Prism 5 software.

### Homology Modelling and Structural Analysis

The dodecameric structure of *Mycobacterium tuberculosis* H37Rv strain dodecin (PDB code 3OQT) (Grininger et al., 2009) was used for the structural analysis of *M.tb* dodecin, and as a template for modeling the BCG Moreau dodecin complex. Comparative modeling was carried out using the software MODELLER 9.18 (Webb and Sali, 2014), and twenty models were generated. To optimize each of the models, we used a protocol of 300 iterations of the built-in Modeller’s conjugate gradient energy minimization algorithm, followed by Modeller’s molecular dynamics routine. For each model, the protocol was repeated two times or until Modeller probability density function returned values greater than 1 × 10^6^. The model with the lowest DOPE score was validated by analysis of the stereochemical properties with the verify3D server (Eisenberg et al., 1997) and inspection of the Ramachandran plot. After validation, the most probable protonation state of the titratable residues at pH 7.0 was calculated for each dodecin complex using the software Propka (Olsson et al., 2011).

The structures of both dodecin complexes were subsequently used for the analysis of the interaction energy between the polypeptide chains within each dodecamer using the INTAA server (Galgonek et al., 2017) with the AMBER parm99 force field (Cheatham et al., 1999). The electrostatic potential surface of each of the structures was analyzed using pymol vacuum electrostatics function, and the dodecin complex of *H. halophila* (PDB code 2VXA) was used for comparison. To investigate if the difference in length could cause any major differences in the flexibility of the complex, we also carried out normal mode analysis (NMA) to understand the overall flexibility of each complex, using an elastic network model (ENM) implemented in the R package Bio3D (Grant et al., 2006). The root-mean-square fluctuations (RMSF) of the residues of each complex were calculated from the normal modes and compared.

## Results

### A single Nucleotide Polymorphism (SNP) Leads to A Shorter N-Terminal Dodecin in BCG Moreau

**Figure 1:**
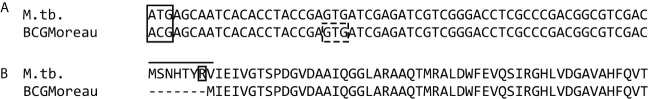
DNA and protein alignment of the dodecin locus. **(A)** Alignment of dodecin coding regions from *M.tb* and *M. bovis* BCG Moreau, showing that a T-C transition (within the solid-line rectangle) leads to the loss of the ATG start codon in BCG and prediction of GTG as the new start codon downstream (dashed-line rectangle). **(B)** Alignment of the resulting proteins showing the effect of this mutation in BCG, with the loss of seven N-terminal amino acids, including Arg7 (boxed), when compared to *M.tb*.

The BCG Moreau dodecin coding gene (BCG_M1530c) carries the same SNP described for the *M. bovis* AF2122-97 and BCG Pasteur orthologs to *M. tuberculosis* H37Rv *rv1498A*. As shown in **Figure 1A**, a T-C transition leads to the loss of the start codon in BCG and the proposition of the following GTG (a known alternative to ATG) as the new start codon, resulting in a protein product with complete sequence homology to the dodecin of *M.tb*, but missing the seven N-terminal amino acids (**Figure 1B**). Among the lost residues it is important to emphasize arginine (Arg7), already described as essential to the *M.tb* dodecin extremophilic characteristics, as it participates in one of the structural stabilizing salt bridges of this complex.

Analysis of other species belonging to the *Mycobacterium tuberculosis* complex shows this SNP to be present only in the animal strains (such as *M. bovis*, *M. bovis* BCG strains and *M. caprae*) and *M. africanum* II (such as *M.tb* variant africanum K85) clades, while the *M.tb* ‘ATG pattern’ is observed throughout the other clades.

Prior to further assays, the cloned *M.tb* H37Rv and *M. bovis* BCG Moreau dodecin genes were re-sequenced to confirm the annotated genotypes on our working samples (data not shown).

### Thermostability, but Not Dodecamerization Capacity, Is Hampered by N-Terminal Loss

**Figure 2:**
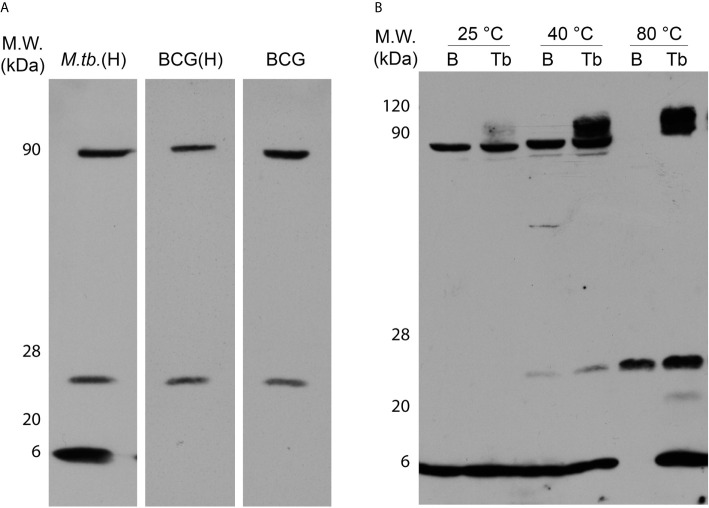
Characterization of *M.tb* (Tb) and BCG Moreau **(B)** recombinant dodecins. Prior to western blotting, 20 µg of proteins from each sample (total proteins from IPTG-induced *E.coli* expressing dodecin clarified lysates) were resolved on 15% SDS-PAGE. We analyzed the effect of the loss of the seven N-terminal amino acids on dodecin **(A)** dodecamerization capacity and **(B)** thermostability. *E. coli* expressing the BCG Moreau dodecin lacking the 6-His tag was included (**A**; BCG). For thermostability assays, protein samples were incubated for 30 min in each condition tested, prior to sample clarification by centrifugation and analysis of supernatant by SDS-PAGE and western blotting. Results are representatives of a set of three independent experiments.

To verify the possible functional impacts of the N-terminal 7-AA loss, BCG Moreau and *M.tb* rDods were obtained in *E. coli*. A mouse polyclonal serum raised against purified rDod-BCG-6His was used in western blotting assays with total proteins from IPTG-induced *E. coli* cultures expressing rDod-BCG or *M.tb*-6HIS, revealing bands corresponding to different oligomerization states - monomer (~7 kDa), tetramer (~27 kDa) and dodecamer (~90 kDa) (**Figure 2A**). This pattern was observed on all samples analyzed, with the exception of the monomer, more detectable on the lysate expressing *M.tb* dodecin and barely on the one expressing the BCG protein. This could be happening due to different equilibrium rates among the distinct oligomerization states between BCG and *M.tb* dodecins, allowing monomer to be more prominent in the *M.tb* sample. Furthermore, similar patterns were detected with the His-tagged and non-tagged BCG proteins, indicating no interference from this tag.

Analysis of protein thermostability shows that while the dodecameric form of the *M.tb* rDod remains in solution after incubation at 80°C, the same is not true for the BCG dodecamer, no longer detected in the soluble fraction above 40°C (**Figure 2B**). Moreover, the pattern of thermostability loss differs between these two rDods, BCG shifting from a dode- (~90 kDa) and monomeric (~7 kDa) mixture at 40°C to an all tetrameric (~27 kDa) soluble form at 80°C, and *M.tb* from a dode- and monomeric mixture at 25°C to a dode-, tetra- and monomeric mixture starting at 40°C, with more equally distributed forms at 80°C.

**Figure 3:**
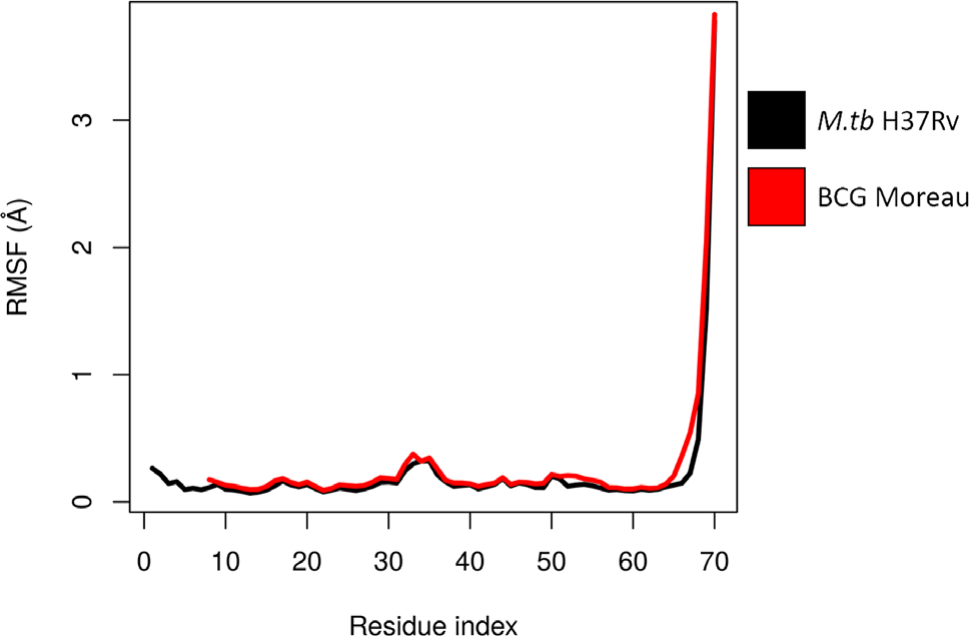
Flexibility of dodecin chain A. The chain A RMSF values calculated from ENM for each complex are shown throughout the protein sequence, suggesting a similar flexibility pattern between *M.tb* and BCG Moreau dodecin complexes.

**Figure 4:**
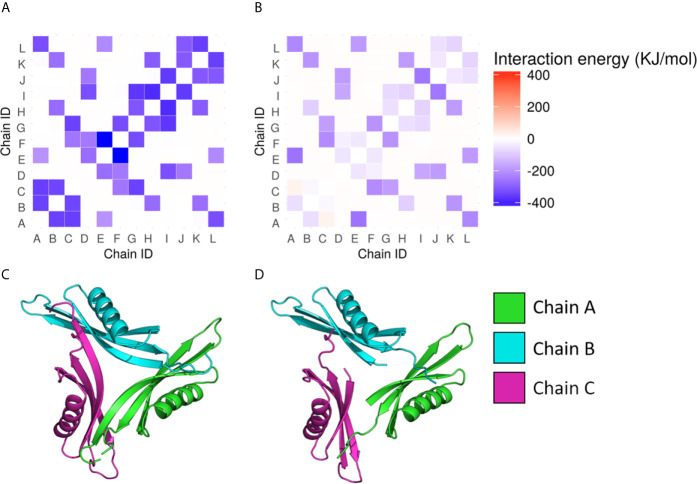
Interchain interaction energy in the dodecamers. The heatmaps in **(A, B)** show respectively the interaction energies between the protein chains in the *M.tb* and the BCG Moreau dodecamers; the energy is depicted as a color scale and the 12 chains of each dodecamer are identified from A to L in both axes. The bottom images **(C, D)** depict one of the faces of the dodecamer of *M.tb* dodecin and BCG Moreau, respectively.

The structures used for the analysis of *M.tb* dodecin (3OQT) and the model of the BCG Moreau complex were both evaluated using the verify3D server and showed respectively more than 89.8% and 83.7% of residues with scores above the threshold. Moreover, *M.tb* dodecin complex showed 99% of the residues in the allowed or favored regions of the Ramchandran plot, while BCG Moreau complex structure showed 98.7%, revealing that both are reliable models. The RMSF from the normal modes suggests that despite being shorter than *M.tb*’s, BCG Moreau dodecin shows no significant differences in flexibility (**Figure 3**). On the other hand, the interaction energy data from the INTAA server shows that the BCG Moreau dodecin (**Figure 4B**) has lower interaction energies between its monomers when compared to the *M.tb* dodecamer (**Figure 4A**), which might explain its reduced thermostability. The structure of the complex suggests that this reduction on interaction energy could be a consequence of the reduced interaction surface caused by the shorter N-terminal segment of the BCG Moreau, as can be seen in one of the faces of the dodecamer represented in **Figures 4C, D**.

### Riboflavin Binding Capacity Is Significantly Decreased for the BCG Dodecamer

**Figure 5:**
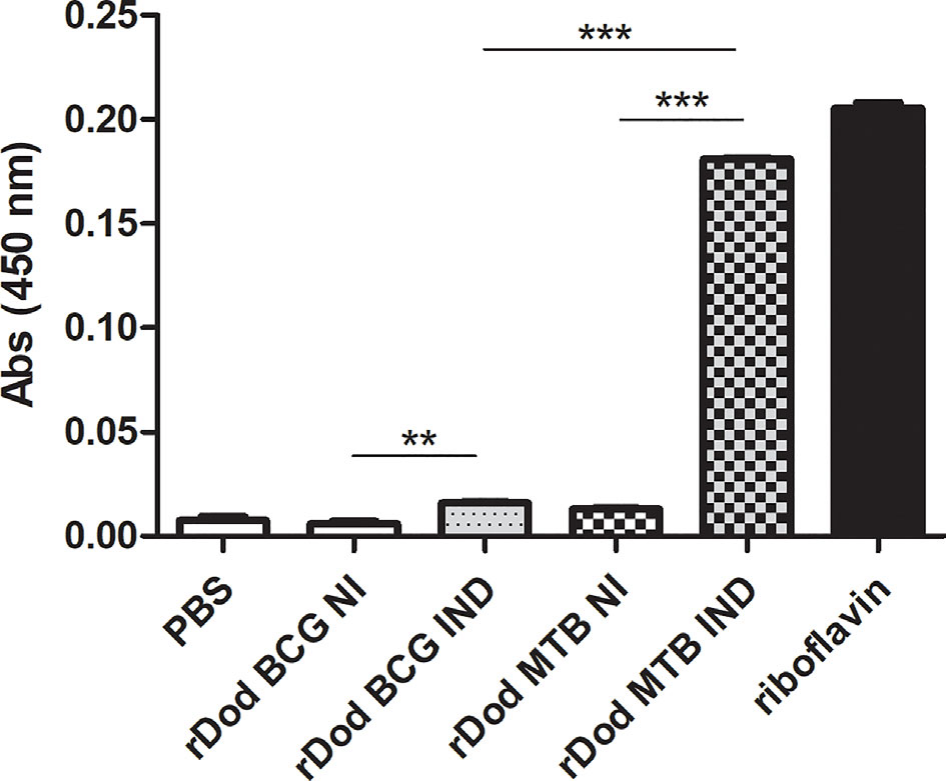
Riboflavin-binding capacity of BCG and *M.tb* dodecin forms. Riboflavin (λ=450nm) detected on 10kDa filter retentate after incubation of saturated riboflavin solution with whole protein extracts from non-induced (NI) or induced (IND) *E. coli* cultures expressing *M.tb* or BCG Moreau (BCG) dodecin genes (rDod). The saturated riboflavin solution was used as maximum value and PBS as a negative control (C-), respectively corresponding to a situation in which all or none of the riboflavin in the input solution bound to dodecin within lysates. Results are represented as the mean +/- SD of a set of three independent experiments. Statistical analysis was performed by unpaired t-test. **p < 0,01, ***p < 0,001.

Regarding dodecin’s known function, it is important to assess its flavin binding capacity to infer possible relevant functional differences between the *M.tb* and BCG forms. To investigate this we performed a column-based assay comparing *E. coli* clarified lysates, expressing or not DOD-BCG or *M.tb*-6HIS. These dodecin-expressing lysates were produced in the same way as those used for the western blotting assays with recombinant proteins. The same amount of total proteins from each respective lysate was loaded per column (500 ug). Proteins with molecular weight greater than 10 kDa were concentrated, washed with PBS and then incubated with a saturated riboflavin solution. Upon interaction and further washing with PBS to remove unbound riboflavin, absorbance at 450nm should represent dodecin-bound flavins on the retentate. As can be seen in **Figure 5**, whole cell lysate of IPTG-induced *E. coli* expressing *M.tb* dodecin binds significantly more riboflavin when compared to the equivalent lysate expressing BCG dodecin. This suggests that the shorter BCG dodecin has a lower riboflavin binding capacity when compared to *M.tb*’s. Significant differences are also detected between the induced and non-induced lysates, indicating that, even though whole cell lysates were used, riboflavin binding is dodecin-specific, as other *E. coli* proteins from non-induced samples could not mimic the results obtained from induced samples. Furthermore, when comparing BCG induced and non-induced samples, a small but significant difference could be detected, indicating that the BCG rDod is still capable of riboflavin binding, but not in the same degree as *M.tb*’s.

**Figure 6:**
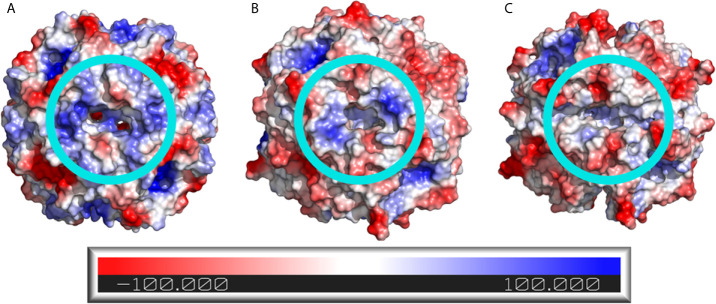
Electrostatic potential surface of the dodecin dodecamers. The electrostatic potential surface of **(A)** *H.* h*alophila*, **(B)** *M.tb*, and **(C)** BCG Moreau dodecins is shown as a color scale in the protein surface, where positively charged regions are colored blue and negatively charged regions are colored red. The light blue circles highlight the riboflavin binding site.

Analysis of the electrostatic potential surface of the dodecamers shows that the predicted BCG Moreau dodecin complex has differences around the riboflavin binding site when compared to the dodecin complexes of *H. halophila* and *M.tb*. Both the *H. halophila* (**Figure 6A**) and *M.tb* (**Figure 6B**) dodecamers display a positively charged cavity in the riboflavin binding pocket, whereas the BCG Moreau variant cavity (**Figure 6C**) has a cleft with less evident positive charges. The BCG Moreau dodecin also displays a different overall shape in its binding pocket, forming a shallow cleft instead of the deeper pocket present in its counterparts, which could explain the difference observed in the binding of riboflavin.

### Native Dodecins Show No Detectable Differences in In-Gel Molecular Weight and Thermostability

**Figure 7:**
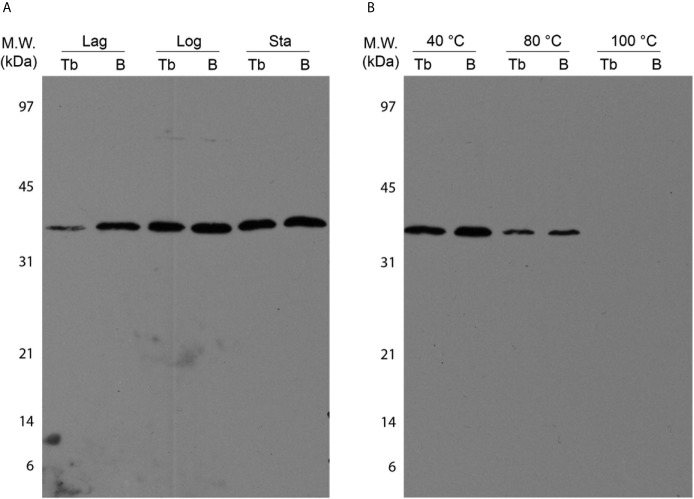
Characterization of *M.tb* (Tb) and BCG Moreau **(B)** native dodecins by western blotting. Prior to western blotting, 20 µg of total proteins from BCG Moreau and *M.tb* clarified lysates were resolved on 15% SDS-PAGE. **(A)** Expression during axenic growth was analyzed on Lag, logarithmic (Log) and stationary (Sta) phases. **(B)** Thermostability assays were performed incubating protein samples at 40, 80 and 100 °C for 30 min prior to sample clarification by centrifugation and subsequent analysis by western blotting. Results are representatives of a set of three independent experiments.

In-gel assays were performed using BCG Moreau and *M.tb* H37Rv whole cell lysates to analyze native dodecin features and compare it to the recombinant protein assays. As shown in **Figure 7A**, dodecin can be detected with an apparent MW corresponding to an hexameric form (~37 kDa) on all axenic growth phases analyzed, with lower intensity bands on the lag phase for both bacteria. Moreover, no differences in the molecular weight of the hexamer can be detected, and this is the only form seen in all native protein assays.

Contrasting with the rDod assays, no differences in the thermostability features were detected between the BCG and *M.tb* native dodecins, and the loss of thermostability shows a similar pattern, shifting from a soluble hexamer observed in both extracts up to 80 °C incubation to a no longer detectable soluble dodecin form at 100 °C (**Figure 7B**).

## Discussion

Despite its almost century old use as the only available vaccine against tuberculosis, there are still several knowledge gaps regarding *M. bovis* BCG physiology, hindering functional comparisons, especially when focusing on the Brazilian vaccine strain, BCG Moreau. One example is dodecin, a protein characterized in *M.tb* and other bacteria, both structurally (Meissner et al., 2007; Liu et al., 2011) and functionally, in relation to its flavin-binding mechanisms (Scheurer et al., 2017; Bourdeaux et al., 2018). Nevertheless, some important questions still remain to be answered regarding its physiological function.

Dodecin has been studied within the Eubacteria, including *M.tb*, *Streptomyces coelicolor* and *S. davawensis* (Ludwig et al., 2018; Bourdeaux et al., 2019). It is already known that the *M.tb* homolog assumes a dodecamer homopolymeric conformation, important for its thermo- and halophilic characteristics, and that these features are related to its tight and cohesive structure, mainly stabilized by salt bridges (Liu et al., 2011). Among the amino acids contributing to these bonds in the *M.tb* homo-olygomer there is an arginine (Arg7), predicted to be lost in the BCG Moreau homolog.

As can be observed from the performed rDod assays, there is a difference in stability between the complexes, with the BCG protein being less thermostable. This could be a direct result of the loss of Arg7, leading to a decrease in the number of salt bridges necessary to stabilize the dodecameric form. In this case, the structure would more readily break down when extra energy (heat) is added to the system or the ionic strength is altered by increasing the salt concentration. The *in silico* analysis shows that the lack of the seven N-terminal residues causes great impact in the interactions between the neighboring chains within the dodecamer, not only due to the lack of the Arg7 residue, but also due to the contribution of interactions involving the other residues in this region. The evidence points to an important role of the *M.tb* dodecin N-terminal loop in maintaining the interaction between the monomers, and that its loss could decrease the stability of the dodecamer, altering the thermostability profile, as observed in the assay with rDods.

Thermophilic proteins are not unusual in the *M.tb* proteome, and some, such as HsaD and TBNAT are essential for the infectious process (Lack et al., 2009), as others, such as EccA1 (part of the ESX-1 secretion system) are known virulence factors (Ganaie et al., 2011). This microbial adaptation is probably due to the temperature rise during the initial infection of immune system cells. From the bacterial perspective, thermophilic molecules that would be involved in these initial steps are advantageous as they could aid the microorganism to surpass this moment and reach later infection states.

As shown by the comparative sequence analysis, the SNP found in the BCG Moreau dodecin gene is common to animal strains and to the *M. africanum* II clade, indicating a recent mutational event, not related to vaccine attenuation. Our results indicate a direct impact of this mutation on the classic function of dodecin, resulting in a decrease in riboflavin binding capacity for the predicted BCG Moreau protein. This finding is in agreement with the structural analysis, showing differences in the flavin-binding cavity opening between both complexes, which could lead to differential flavin access to the binding residues, mainly Trp39 (from Rv1498A). This decreased flavin binding capacity could be due to higher solvent accessibility, leading to an unstable interaction between flavin and its binding residues. This phenotype observed for BCG dodecin would speak against the genotype fixation throughout clades of the *Mycobacterium tuberculosis* complex, as a less functional protein could diminish bacterial fitness during evolution. But it is important to point out that only dodecin’s flavin-binding capacity was evaluated in this work, so we cannot rule out other possible yet unknown functions for this complex, interfering on BCG dodecin fitness. Although we also tried to perform this column-based riboflavin-binding assay with native lysates, no significant signal could be detected (data not shown), probably due to low dodecin expression.

Western blot analysis of native dodecins present in whole cell lysates identifies a band with molecular weight compatible with the hexameric form, with similar molecular weight and thermostability profile between *M.tb* and BCG. Even though a 1kDa difference in the molecular weight of the monomer would be undetectable by SDS-PAGE, such difference should be apparent in the hexameric form (~37 kDa). Moreover, there is no distinction in the thermostability profile between the native *M.tb* and BCG dodecins, contrary to what was observed in assays employing recombinant proteins.

It is important to stress that throughout our in-gel assays with recombinant dodecins several oligomerization states (monomer, tetramer and dodecamer) could be detected. But only the hexameric form was evident when using native proteins. Other studies show that the dodecameric form exists in solution and can be detected by techniques such as analytical ultracentrifugation, but it breaks down to the hexamer upon SDS-PAGE analysis (Meissner et al., 2007; Liu et al., 2011). As already known, the dodecin dodecamer can be stabilized by interactions with free flavins, such as FMN, so differences in the intracellular concentrations of these molecules between *E. coli* and *M. bovis* BCG Moreau could explain our contrasting results regarding recombinant and native dodecins, indicating that it may be due to an experimental artifact (Liu et al., 2011; Bourdeaux et al., 2018).

Our data could suggest that even with the SNP affecting the start codon, BCG Moreau dodecin is still being produced in a similar way to *M.tb*, as shown by our western blotting assays with native proteins. A possible explanation for the different results with recombinant and native proteins would be that ACG functions as an alternative start codon in Mycobacteria, as already described for other organisms, such as for the alanyl-tRNA synthetase gene from *Saccharomyces cerevisiae* (Tang et al., 2004), where it was shown that its use leads to the production of a polypeptide different from the canonical one encoded by this yeast mitochondrial gene. To verify this hypothesis, further experiments should be performed to confirm ACG as an alternative translational start site in the mycobacterial context, since only ATG, GTG and TTG have already been characterized as such in *M.tb* (DeJesus et al., 2013).

Our results contribute to a better understanding of the molecular mechanisms involved in extremophilic features, as those described for *M.tb* dodecin. We show that the loop formed by the seven N-terminal amino acids is essential for the protein’s thermophilic features, mainly due to the enhancement of subunit interactions, possibly for example, by the salt bridge involving Arg7. But this phenotype, observed for the recombinant predicted BCG protein, may not be representative of the real BCG physiology as the native protein behaves similarly to *M.tb* dodecin. This could result from selective forces acting on this locus and the use of an alternative start codon, maintaining protein features even with the T-C transition genotype. In this context, the possible role of ACG as a real mycobacterial translational start codon still needs to be verified employing, for example, reporter gene assays. Further functional and structural characterization of the native BCG Moreau protein is needed, in order to better understand the impact of this mutation on bacterial fitness, intracellular survival and vaccine features.

## Data Availability Statement

The original contributions presented in the study are included in the article/supplementary material. Further inquiries can be directed to the corresponding author.

## Ethics Statement

The animal study was reviewed and approved by Commission for the Use of Laboratory Animals from IOC, Fiocruz (License CEUA/IOC L-020/2016).

## Author Contributions

MS, PC and LM-L conceived and designed all experimental assays. LM and AG designed and performed *in silico* structural analysis. MS, BL, PC, LM and AS-G performed the experiments. All authors analyzed the data. LM-L and AG contributed to the reagents/materials/analysis tools. MS and LM-L wrote the paper. All authors contributed to the article and approved the submitted version.

## Funding

This work was funded by the Oswaldo Cruz Foundation and the Brazilian National Council for Scientific and Technological Development (project CNPq-PAPES VI 421923/2017-2). This study was financed in part by the Coordination for the Improvement of Higher Education Personnel (Coordenação de Aperfeiçoamento de Pessoal de Nível Superior - CAPES) - Finance Code 001.

## Conflict of Interest

The authors declare that the research was conducted in the absence of any commercial or financial relationships that could be construed as a potential conflict of interest.
